# Sleep hygiene behaviours mediate the association between health/e‐health literacy and mental wellbeing

**DOI:** 10.1111/hex.13837

**Published:** 2023-08-07

**Authors:** Mehran Alijanzadeh, Rafat Yahaghi, Jalal Rahmani, Nahid Yazdi, Elahe Jafari, Hashem Alijani, Narges Zamani, Razie Fotuhi, Elham Taherkhani, Zeinab Buchali, Masoume Zarenejad, Narges Mahmoudi, Najmeh Shahmahdi, Leila Poorzolfaghar, Safie Ahmadizade, Azam Shahbazkhania, David Gozal, Chung‐Ying Lin, Amir H. Pakpour

**Affiliations:** ^1^ Social Determinants of Health Research Center, Research Institute for Prevention of Non‐Communicable Diseases Qazvin University of Medical Sciences Qazvin Iran; ^2^ Department of Child Health University of Missouri School of Medicine Columbia Missouri USA; ^3^ Institute of Allied Health Sciences, College of Medicine National Cheng Kung University Tainan Taiwan; ^4^ Biostatistics Consulting Center, National Cheng Kung University Hospital, College of Medicine National Cheng Kung University Tainan Taiwan; ^5^ Department of Public Health, College of Medicine National Cheng Kung University Tainan Taiwan; ^6^ Department of Occupational Therapy, College of Medicine National Cheng Kung University Tainan Taiwan; ^7^ Department of Nursing, School of Health and Welfare Jönköping University Jönköping Sweden

**Keywords:** e‐health, health literacy, Iran, mental wellbeing, sleep behaviour

## Abstract

**Background:**

Health literacy and e‐health literacy are important factors helping people shape awareness of health behaviours in different aspects, including sleep hygiene behaviours. Good sleep hygiene behaviours promote sleep quality and are beneficial to overall mental wellbeing.

**Objective:**

We aimed to examine if sleep hygiene behaviours may mediate the association between health literacy/e‐health literacy and mental wellbeing.

**Methods:**

Adult Iranian subjects (*n* = 9775; mean [SD] age = 36.44 [11.97] years; 67.3% females) completed the Health Literacy Instrument for Adults, eHealth Literacy Scale, three items on sleep hygiene behaviour that have been used in prior research and the Short Warwick Edinburgh Mental Wellbeing Scale. Data were then subjected to structural equation modelling (SEM) including 500 bootstrapping resampling to examine whether sleep hygiene is a mediator in the relationship between health literacy/e‐health literacy and mental wellbeing.

**Findings:**

Both health literacy and e‐health literacy were significantly associated with mental wellbeing (*r* = .63 for health literacy and .39 for e‐health literacy; *p* < .001) and sleep hygiene behaviours (*r* = .58 for health literacy and .36 for e‐health literacy; *p* < .001). Sleep hygiene behaviours were significantly associated with mental wellbeing (*r* = .42; *p* < .001). Moreover, SEM that incorporated bootstrapping approaches indicated that sleep hygiene behaviours were significant mediators in the association between health literacy/e‐health literacy and mental wellbeing.

**Conclusions:**

We conclude that health literacy and e‐health literacy are associated with mental health wellbeing in the Iranian population. Additionally, the association could be mediated via sleep hygiene behaviours.

**Patient or Public Contribution:**

The study was co‐designed with healthcare providers from the vice‐Chancellor's Office for Health Affairs of Qazvin University of Medical Sciences as equal partners. Moreover, the women's health volunteers were involved in the design of the study.

## 1 INTRODUCTION

Health literacy is an important element playing a role in protecting people from illness as it facilitates the adoption of healthy behaviours thereby achieving effective prevention of disease conditions.^1^, ^2^ Specifically, when good health literacy is present, knowledge regarding how to improve health and subsequently engage in healthy behaviours while avoiding unhealthy behaviours is more likely to occur.^3^ Therefore, the World Health Organization has clearly emphasized the importance of health literacy for human health, and further delineated a definition for health literacy; that is, whether an individual has the ability to obtain, interpret and use information to promote and maintain good health.^4^

In addition to traditional health literacy (i.e., having health literacy via traditional resources such as newspapers and TV), the technological advances that have occurred more recently have extended health literacy into an e‐format, now denominated as e‐health literacy.^5^ e‐health literacy is defined as ‘the ability to gather and appropriately process health information retrieved online’, such as information from *Google* engine and Facebook posts.^6^ In this regard, people having good e‐health literacy need to have certain levels of skills in using the internet. Nevertheless, having good e‐health literacy is considered as having similar effects to having good traditional health literacy, and therefore is a major contributor to improving health‐related behaviours and promoting health. For example, e‐health literacy has been associated with people's knowledge and health behaviours (i.e., screening practice) in colorectal cancer and regular screening practice behaviours.^7^ Similarly, a longitudinal study found that e‐health literacy is protective for aging adults with heart failure in the prevention of cardiac events via enhancement of several mediators, including reduced insomnia, decreased psychological distress and improved medication adherence ^8^ Moreover, ample evidence has shown that health literacy is associated with a good sleep because of the improved health behaviours.^9^, ^10^ In brief, both e‐health literacy and traditional health literacy are important for engagement of health behaviours that lead to better health outcomes, including sleep.

Health literacy and e‐health literacy could help improve people engaging in good sleep hygiene behaviours, and sleep hygiene behaviour is one of the behaviours that is critically important for mental wellbeing is sleep hygiene.^11^, ^12^, ^13^, ^14^ Specifically, adherence to good sleep hygiene behaviours promotes better sleep,^15^, ^16^, ^17^ which in turn is crucial and essential for health promotion in a variety of multifaceted domains. Good sleep may facilitate optimal concentration and focus during the performance of daytime activities (e.g., working or studying),^18^, ^19^ may promote human development both biologically and neurologically,^20^, ^21^ and has been ascribed as a major factor enabling good mental health.^22^, ^23^, ^24^ Thus, when adherence to satisfactory sleep hygiene behaviours, may enhance good sleep which may consequently maintain or improve health, including mental wellbeing.

Notwithstanding the aforementioned evidence and studies examining the associations between healthy literacy, sleep and mental health,^12^, ^25^ rarely have studies used inferential statistics to examine the mediation roles of sleep hygiene in the association between health literacy and mental wellbeing. An improved understanding of the contribution of sleep hygiene to mental wellbeing can be helpful for healthcare providers to design appropriate health‐promoting programs. If sleep hygiene behaviours are significant mediators of mental wellbeing via improved health literacy, healthcare systems can launch educational campaigns aimed at improving health literacy in the general population. In parallel, healthcare providers may design and implement different programs to improve sleep hygiene behaviours via behavioural change techniques, occupational therapy processes or health behaviour theories.^26^, ^27^, ^28^ Accordingly, sleep hygiene behaviours in the general population could be improved and consequently elevate their mental wellbeing.

The present study aimed to examine the mediator roles of sleep hygiene in the association between two types of health literacy (i.e., traditional healthy literacy [hereafter referred to ‘health literacy’] and e‐health literacy) and mental wellbeing. Moreover, we hypothesized that (i) sleep hygiene behaviours are significant mediators in the association between traditional health literacy and mental wellbeing and (ii) sleep hygiene behaviours are significant mediators in the association between e‐health literacy and mental wellbeing.

## 2 METHODS

### 2.1 Participants and data collection

This study is part of a broader study focused on health literacy and mental wellbeing amongst Iranian citizens residing in the city of Qazvin.^29^, ^30^ Data were collected using a stratified cluster sampling technique from 70 strata defined by postal codes within the Qazvin province. A team of 25 trained research associates employed a random selection method to choose families from the existing list of families associated with health centres within each stratum. Paper‐and‐pencil questionnaires were used to gather all research‐related measures from adult subjects. All participants provided written informed consent before starting the survey.^29^ The Ethics Committee of Qazvin University of Medical Sciences (protocol code: IR.QUMS.REC.1400.225) approved the study.

### 2.2 Measures

#### 2.2.1 Health Literacy Instrument for Adults (HELIA)

The HELIA is a 33‐item questionnaire administered via self‐report. Amongst the 33 items, five domains are defined: reading (4 items), access to information (6 items), understanding (7 items), appraisal (4 items) and decision‐making/behavioural intention (12 items).^31^ Sample items of HELIA included ‘Reading educational materials about health (booklets, pamphlets, leaflets) is easy for me (reading)’, ‘I can find health information about a specific disease when I need to (access to information)’, ‘I can understand signage guidelines in hospitals, clinics and health centers (understanding)’, ‘I can communicate trusted health information to others (appraisal)’ and ‘I am health‐conscious in any situation (decision making/behavioural intention)’. All items are rated on a 5‐point Likert scale ranging from 1 (never) to 5 (always). The domain scores are then linearly converted into a 0–100 scale (i.e., score 1 converted into 0; 2 into 25; 3 into 50; 4 into 75 and 5 into 100). A higher score in the HELIA indicates better health literacy. Moreover, prior evidence shows that the HELIA is psychometrically sound in its Persian language when administered to Iranians.^29^

#### 2.2.2 eHealth Literacy Scale (eHEALS)

The eHEALS is an eight‐item questionnaire administered via self‐report. Amongst the eight items, one domain of e‐health literacy has been validated.^32^, ^33^ A sample item is ‘I know how to find helpful health resources on the Internet’. All items are rated on a 5‐point Likert scale ranging from 1 (strongly disagree) to 5 (strongly agree). A higher score in the eHEALS indicates better e‐health literacy. Moreover, the eHEALS is psychometrically robust in the Persian language.^8^, ^34^

#### 2.2.3 Sleep hygiene behaviour measure

Three items were used to assess sleep hygiene behaviours: (i) How many times did you make your bedroom a restful place over the past week? (ii) How many times did you avoid going to bed feeling hungry or thirsty? (iii) How many times did you avoid anxiety and stress‐provoking activities before going to bed? All items are rated on a 5‐point Likert scale ranging from 1 (never) to 5 (always). Then, the three items were averaged to indicate sleep hygiene behaviours. A higher score in the sleep hygiene behaviours measure indicates better sleep hygiene. Moreover, these three sleep hygiene behavioural items in the Persian language showed satisfactory properties in their internal consistency and criterion‐related validity with the Pittsburgh Sleep Quality Index.^35^

#### 2.2.4 Short Warwick Edinburgh Mental Wellbeing Scale (SWEMWS)

The SWEMWS is a seven‐item questionnaire administered via self‐report. Amongst the seven items, one domain of mental wellbeing has been validated.^36^ All items are rated on a 5‐point Likert scale ranging from 1 (never) to 5 (always). A higher score in the SWEMWS indicates better mental wellbeing. The SWEMWS has been psychometrically validated in the Persian language.^37^

#### 2.2.5 Demographic measures

All participants provided information on their age, gender, educational status, residence and marital status.

### 2.3 Data analysis

Demographics were initially presented using descriptive statistics (i.e., mean with SD and frequency with percentage). Pearson correlations and descriptive statistics were then used to examine the associations and central tendencies of the studied variables (i.e., HELIA domains, eHEALS, sleep hygiene behaviours and mental wellbeing). Finally, three models were constructed via structural equation modelling (SEM) to examine the mediation effects of sleep hygiene behaviours in the association between health/e‐health literacy and mental wellbeing.

**Figure 1:**
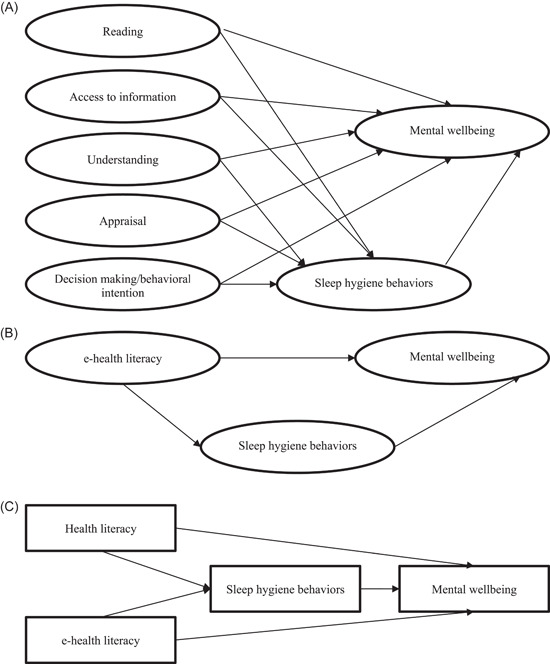
Conceptual model of the proposed models. (A) The mediation model of sleep hygiene behaviours being a mediator in the associations between separated traditional health literacy domains and mental wellbeing. (B) The mediation model of sleep hygiene behaviours being the mediator in the association between e‐health literacy and mental wellbeing. (C) The mediation model of sleep hygiene behaviours being the mediator in the association between integrated health literacy (i.e., including traditional health literacy and e‐health literacy) and mental wellbeing. Latent constructs are represented using circles; observed constructs using rectangles.

In the first model, mediation effects of sleep hygiene behaviours were tested in the association between health literacy (assessed via HELIA with separated domains) and wellbeing (Figure 1A). In the second model, mediation effects of sleep hygiene behaviours were tested in the association between e‐health literacy (assessed via eHEALS) and wellbeing (Figure 1B). In the third model, the mediation effects of sleep hygiene behaviours were tested in the associations of health literacy and e‐health literacy with wellbeing (Figure 1C). Moreover, for the first and the second models, all the factors (i.e., health literacy domains, e‐health literacy, sleep hygiene behaviours and mental wellbeing) were treated as latent constructs to achieve take care of the measurement issues in the models. However, for the third model, two submodels were tested: the first submodel treated all the factors as latent variables and the second submodel treated all the factors as observed variables. The three models were tested because they provide different information: the first model provides information for specific domains of health literacy; the second model provides information for e‐health literacy and the third model provides information about health literacy and e‐health literacy simultaneously and treating the health literacy as a whole.

For all the SEM models, age, gender, residence and educational status were controlled. Moreover, the following fit indices were used to examine if the first and the second models are supported: comparative fit index (CFI) > 0.9, Tucker–Lewis index (TLI) > 0.9, root mean square error of approximation (RMSEA) < 0.08 and standardized root‐mean‐square residual (SRMR) < 0.08.^38^, ^39^, ^40^ The third model was a saturated model and did not have fit indices. Nevertheless, if the first and the second models have satisfactory fit indices, the third model could be considered as supported as well. When the models were supported, the mediation effects of sleep hygiene behaviours were further assessed using the bootstrapping methodology.^41^ As such, when the 95% confidence interval (CI) of the bootstrapping resamples (there were 500 resamples used for each model in the present study) does not cover 0, the mediation effects are considered to be significant.^42^

## 3 RESULTS

A total of 9775 subjects completed the surveys. Their mean [SD] age was 36.44 [11.97] years and 67.3% were females (*n* = 6576) with 1.3% not indicating gender. Nearly half of the participants were residing in Qazvin (*n* = 4655; 47.6%) and 74.6% lived in a city (*n* = 7287). Most of the participants had completed higher education (32.9% had high school diplomas and 39.4% had university degrees) and 71.5% were currently married (Table 1).

**Table 1:** Overall characteristics of the participants (*n* = 9775).

	Mean ± SD or *n* (%)
Age (years)	36.44 ± 11.97
Gender	
Male	3073 (31.4)
Female	6576 (67.3)
Missing	126 (1.3)
City	
Qazvin	4655 (47.6)
Takestan	1216 (12.4)
Avaj	330 (3.4)
Abyek	657 (6.7)
Bueenzahra	963 (9.9)
Alborz	1867 (19.1)
Missing	87 (0.9)
Educational status	
Primary school	1082 (11.1)
Secondary school	1576 (16.1)
Diploma	3220 (32.9)
University	3851 (39.4)
Missing	46 (0.5)
Residence	
City	7287 (74.6)
Rural	2309 (23.6)
Missing	179 (1.8)
Marital status	
Single	1779 (18.2)
Married	6987 (71.5)
Divorced/widowed	90 (0.9)
Missing	919 (9.4)

Table 2 shows the correlations and descriptive statistics of the variables included in the study. Specifically, sleep hygiene behaviours were significantly associated with all the HELIA domains in a moderate to strong magnitude positively (*r* = .47–.52; *p*< .001); and significantly associated with e‐health literacy in a moderate magnitude positively (*r* = .36; *p* < .001). Similarly, mental wellbeing was significantly associated with all the HELIA domains in a moderate to strong magnitude positively (*r* = .49–.56; *p* < .001) and significantly associated with e‐health literacy in a moderate magnitude positively (*r* = .39; *p* < .001). Moreover, sleep hygiene behaviours were significantly associated with mental wellbeing positively (*r* = .42; *p* < .001).

**Table 2:** Pearson correlations and mean (SD) of the studied variables.

	*r*									Mean (SD)
	HELIA_Re	HELIA_Ac	HELIA_Un	HELIA_Ap	HELIA_De	HELIA_TOTAL	SHB	eHealth	MW	
HELIA_Re	–	.67	.68	.61	.50	.82	.49	.47	.49	73.24 (23.23)
HELIA_Ac	–	–	.74	.71	.59	.88	.52	.62	.53	71.76 (22.48)
HELIA_Un	–	–	–	.76	.66	.90	.50	.52	.56	78.94 (20.54)
HELIA_Ap	–	–	–	–	.66	.88	.52	.50	.55	72.45 (22.70)
HELIA_De	–	–	–	–	–	.79	.47	.43	.54	73.20 (19.59)
HELIA_TOTAL	–	–	–	–	–	–	.58	.60	.63	73.87 (18.48)
SHB	–	–	–	–	–	–	–	.36	.42	3.25 (0.96)
eHealth	–	–	–	–	–	–	–	–	.39	31.44 (6.66)
MW	–	–	–	–	–	–	–	–	–	21.90 (4.70)

*Note*: All *p* < .001.

Abbreviations: Ac, access to information; Ap, appraisal; De, decision making/behavioural intention; eHealth, electronic health literacy; HELIA, Health Literacy Instrument for Adults; MW, mental wellbeing; Re, reading; SHB, sleep hygiene behaviours; TOTAL, total score; Un, understanding.

**Figure 2:**
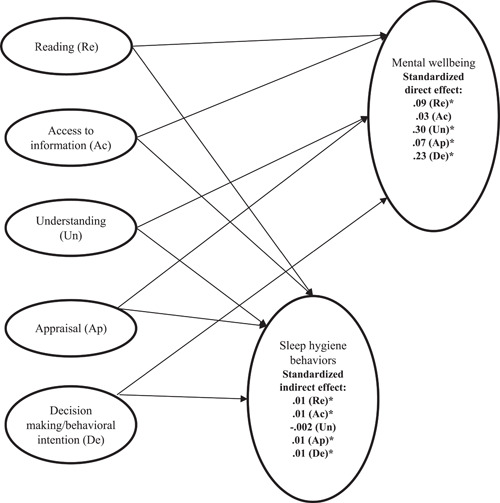
Results of the mediation model of sleep hygiene behaviours being mediator in the associations between separated traditional health literacy domains and mental wellbeing. Model fit: *χ* ^2^ (*df*) = 22,593.172 (973); *p* < .001; standardized root‐mean‐square residual = 0.04; root mean square error of approximation (90% confidence interval) = 0.05 (0.05–0.05); Tucker–Lewis index = 0.92; comparative fit index = 0.93. *p < .05.

**Figure 3:**
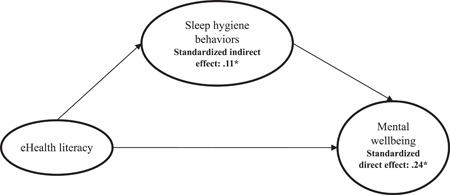
Results of the mediation model of sleep hygiene behaviours being the mediator in the association between e‐health literacy and mental wellbeing. Model fit: *χ* ^2^ (*df*) = 7066.205 (192); *p* < .001; standardized root‐mean‐square residual = 0.04; root mean square error of approximation (90% confidence interval) = 0.06 (0.06–0.06); Tucker–Lewis index = 0.93; comparative fit index = 0.94. *p < .05.

Two proposed mediation models using latent constructs (one for separated HELIA domains and another for eHEALS) had satisfactory fit statistics. For HELIA (Figure 2): *χ* ^2^ (*df*) = 22593.172 (973), *p* < .001, SRMR = 0.04, RMSEA (90% CI) = 0.05 (0.05–0.05), TLI = 0.92 and CFI = 0.93; for eHEALS (Figure 3): *χ* ^2^ (*df*) = 7066.205 (192), *p* < .001, SRMR = 0.04, RMSEA (90% CI) = 0.06 (0.06–0.06), TLI = 0.93 and CFI = 0.94. Furthermore, sleep hygiene behaviours were found to be a positive and significant mediator in the following paths: (i) from HELIA reading domain to mental wellbeing (standardized indirect effect [*β*] = .01; 95% bootstrapping CI = 0.01, 0.02), (ii) from HELIA access to information domain to mental wellbeing (*β* = .01; 95% bootstrapping CI = 0.01, 0.02), (iii) from HELIA appraisal domain to mental wellbeing (*β* = .01; 95% bootstrapping CI = 0.01, 0.02), (iv) from HELIA decision making/behavioural intention domain to mental wellbeing (*β* = .01; 95% bootstrapping CI = 0.004, 0.01) and (v) from eHEALS to mental wellbeing (*β* = .11; 95% bootstrapping CI = 0.10, 0.13). Also, health literacy domains (*β* [*p*] = .09 [<.001] for reading; .30 [<.001] for understanding; .07 [.009] for appraisal and .23 [<.001] for decision making/behavioural intention), except for access to information (*p* = .15) and e‐health literacy (*β* [*p*] = .24 [<.001]) had significantly direct effects on mental wellbeing (Table 3).

**Table 3:** Mediation effects of sleep hygiene behaviours (SHB) in the association between health literacy and mental wellbeing (MW).

Hypothesized path	Standardized direct effect (SE)/*p* value	Standardized indirect effect (SE)/*p* value	95% LLCI, ULCI of indirect effect	Standardized total effect (SE)
Health Literacy Instrument for Adults				
Re → SHB → MW	0.09 (0.02)/<.001*	0.01 (0.003)/<.001*	0.005, 0.016	0.10 (0.02)
Ac → SHB → MW	0.03 (0.02)/.15	0.01 (0.003)/<.001*	0.005, 0.017	0.04 (0.02)
Un → SHB → MW	0.30 (0.03)/<.001*	−0.002 (0.002)/.32	−0.006, 0.0004	0.30 (0.03)
Ap → SHB → MW	0.07 (0.03)/.009*	0.01 (0.004)/.001*	0.007, 0.022	0.08 (0.03)
De → SHB → MW	0.23 (0.02)/<.001*	0.01 (0.002)/<.001*	0.004, 0.012	0.23 (0.02)
eHealth Literacy Scale				
eHealth → SHB → MW	0.24 (0.01)/<.001*	0.11 (0.01)/<.001*	0.10, 0.13	0.35 (0.01)

*Note*: The indirect effects were assessed using 500 bootstrapping resamples. Age, gender, accommodation and education were controlled in all the hypothesized testing.

Abbreviations: Ac, access to information; Ap, appraisal; De, decision making/behavioural intention; eHealth, electronic health literacy; LLCI, lower limit confidence interval; Re, reading; ULCI, upper limit confidence interval; Un, understanding.

* *p* < .05.

**Figure 4:**
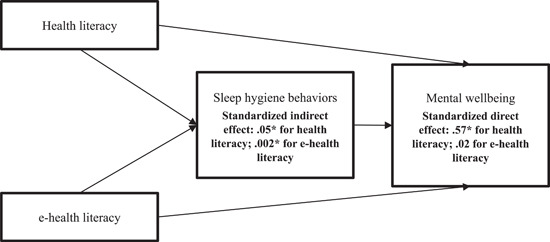
Results of the mediation model of sleep hygiene behaviours being a mediator in the association between integrated health literacy (i.e., including traditional health literacy and e‐health literacy) and mental wellbeing.

Moreover, in the third model, the model with latent variables (i.e., treating all factors as latent) could not be identified [*χ* ^2^ (*df*) = 104,420.76 (190), *p* < .001, RMSEA (90% CI) = 0.237 (0.236–0.238), TLI = 0 and CFI = 0] (detailed information please see Appendix A). Therefore, we proceeded with the second submodel (i.e., using observed variables instead of latent constructs for the factors to satisfy the principle of parsimony in SEM). The integrating HELIA as a whole and eHEALS simultaneously, sleep hygiene behaviours were found to be a positive and significant mediator in the associations of health literacy (*β* = .05; 95% bootstrapping CI = 0.04, 0.06) and e‐health literacy (*β* = .002; 95% bootstrapping CI = 0.000, 0.004) with mental wellbeing (Table 4). In addition, health literacy (*β* = .57; *p* < .001) but not e‐health literacy (*β* = .02; *p* = .09) had significantly direct effects on mental wellbeing (Figure 4).

**Table 4:** Mediation effects of sleep hygiene behaviours (SHB) in the association between health literacy and mental wellbeing (MW).

Hypothesized path	Standardized direct effect (SE)/*p* value	Standardized indirect effect (SE)/*p* value	95% LLCI, ULCI of indirect effect	Standardized total effect (SE)
Traditional health literacy → SHB → MW	0.57 (0.01)/<.001*	0.05 (0.01)/<.001*	0.04, 0.06	0.62 (0.01)
eHealth → SHB → MW	0.02 (0.01)/.09	0.002 (0.001)/.045*	0.000, 0.004	0.02 (0.01)

*Note*: The indirect effects were assessed using 1000 bootstrapping resamples. Age, gender, accommodation and education were controlled in all the hypothesized testing.

Abbreviations: eHealth, electronic health literacy; LLCI, lower limit confidence interval; ULCI, upper limit confidence interval.

* *p* < .05.

## 4 DISCUSSION

The present study used a large Iranian sample to examine if sleep hygiene behaviours mediate the association between health literacy/e‐health literacy and mental wellbeing. Our findings suggest that all domains of health literacy, except for the understanding domain, and e‐health literacy were positively and significantly associated with mental wellbeing via sleep hygiene behaviours. Moreover, all health literacy domains and e‐health literacy were positively and significantly associated with sleep hygiene behaviours and with mental wellbeing. In addition, sleep hygiene behaviours were also positively and significantly associated with mental wellbeing and emerged as a mediator in the relationship between mental well‐being and health literacy. Thus, it is reasonable to assume that any improvements in health literacy may promote people's sleep hygiene behaviours, and subsequently enhance their mental wellbeing.

The associations found between health literacy and sleep hygiene behaviours in the present study concur with prior findings. Indeed, improved health literacy may reduce the prevalence and severity of sleep disorders,^9^, ^10^, ^12^, ^25^, ^43^ as further suggested by the fact that people with lower levels of health literacy also exhibit poorer sleep duration and quality and higher frequency of sleep disorders.^9^, ^25^ In another study by Bonuck et al., health literacy (especially sleep health literacy) was positively associated with healthy sleep.^44^ Thus, the gained knowledge for people to engage in health behaviours (i.e., sleep hygiene behaviours in the present study) may account for improved outcomes (i.e., mental wellbeing in the present study). Indeed, when people know the benefits of any specific health behaviour (i.e., good health literacy), they may generally have improved motivation to adhere to these beneficial behaviours. However, to the best of our knowledge, most prior evidence only examined the association between health literacy and sleep hygiene behaviours, with no studies evaluating the association between e‐health literacy and sleep hygiene. Current findings thus expand on these associations from traditional health literacy to e‐health literacy, suggesting that a disposition to explore electronic sources of information may be equivalent to the previous concepts regarding traditional literacy or even provide incremental value.^45^, ^46^ In the context of our specific focus on sleep hygiene, the presence of increased ability and propensity to obtain knowledge online implied the corresponding tendency to have healthy sleep hygiene behaviours.

The associations found between sleep hygiene behaviours and mental wellbeing in the present study may be ascribable to improved sleep as facilitated by sleep hygiene. Specifically, prior evidence shows that enhanced sleep hygiene behaviours are associated with quality sleep,^15^, ^16^, ^17^, ^47^ and a good night's sleep is associated with good health, including favourable effects on mental wellbeing.^14^, ^22^, ^23^, ^24^ Moreover, a recent study of 815 participants showed that sleep problems were significantly associated with psychological distress (subjects with sleep problems were fourfold more likely to develop anxiety symptoms, and >threefold more likely to have depressive symptoms.^25^ Therefore, improved sleep hygiene behaviours could be associated with better mental wellbeing, while poorer sleep hygiene behaviours might enhance the likelihood of poorer mental health.

In addition, as shown herein, both health literacy and e‐health literacy may improve mental wellbeing as a result of better sleep hygiene behaviours.^44^ The mediation effects tested in the present study revealed that the relationship of health literacy and e‐health literacy with sleep hygiene behaviours could ameliorate or preserve mental wellbeing. We postulate that when individuals have good health literacy or e‐health literacy, they are more likely to effectively search for information related to sleep online or offline and to acquire and implement better knowledge of sleep issues. Such improved knowledge of sleep is likely to encourage individuals to engage in good sleep hygiene behaviours, which in turn results in good sleep. A good night's sleep may then promote health and mental wellbeing.^22^, ^23^, ^24^

There are some limitations in the present study that deserve mention. First, the present study collected data using self‐reports. Therefore, the present findings are subject to several biases related to this type of data collection method, namely single rater bias, recall bias and social desirability bias. Second, given that health literacy could be different between Western and Eastern countries and the present study only recruited Iranians, the present findings may not be generalizable. Future studies are thus needed to examine if the present findings can be replicated in other countries. Third, the measure of sleep hygiene behaviours was not strong. Specifically, we only used three items to assess sleep hygiene behaviours; thus, they might not capture the whole construct of sleep hygiene behaviours. Future studies are thus suggested using other standardized and validated instruments on sleep hygiene behaviours (e.g., Sleep Hygiene Index).^48^ Finally, this was a cross‐sectional study with no intervention such that causal inferences are precluded.

## 5 CONCLUSIONS

In summary, sleep hygiene behaviours emerged as a significant mediator in the association between health/e‐health literacy and mental wellbeing in a large sample of Iranians. These findings indicate the importance of improving health literacy and e‐health literacy in the population, whereby improved health literacy correspondingly ameliorates sleep hygiene behaviours leading to better preservation of mental wellbeing. However, future experimental studies are needed to examine if improving health literacy and e‐health literacy could increase sleep hygiene behaviours, and promote mental wellbeing.

## AUTHOR CONTRIBUTIONS

**Mehran Alijanzadeh, Rafat Yahaghi** and **Amir H. Pakpour**: Conceptualization. **Mehran Alijanzadeh, Rafat Yahaghi** and **Amir H. Pakpour**: Methodology. **Jalal Rahmani, Nahid Yazdi, Elahe Jafari, Najmeh Shahmahdi** and **Hashem Alijani**: Software. **Mehran Alijanzadeh, Rafat Yahaghi** and **Amir H. Pakpour**: Validation. **Amir H. Pakpour**: Formal analysis **Narges Zamani, Rafat Yahaghi, Razie Fotuhi, Masoume Zarenejad, Elham Taherkhani, Zeinab Buchali, Narges Mahmoudi, Leila Poorzolfaghar, Safie Ahmadizade, Azam Shahbazkhania** and **Mehran Alijanzadeh**: Investigation. **Amir H. Pakpour**: Resources. **Chung‐Ying Lin** and **Amir H. Pakpour**: Data curation. **Chung‐Ying Lin** and **Amir H. Pakpour**: Writing—original draft preparation. **Amir H. Pakpour, David Gozal**: Writing—review and editing. **Chung‐Ying Lin**: Visualization. **Amir H. Pakpour**: Supervision **Rafat Yahaghi, Mehran Alijanzadeh, Zeinab Buchali, Jalal Rahmani, Hashem Alijani** and **Amir H. Pakpour**: Project administration. **Amir H. Pakpour**: Funding acquisition. All authors read and agreed to the published version of the manuscript.

## CONFLICT OF INTEREST STATEMENT

The authors declare no conflict of interest.

## ETHICS STATEMENT

This research was reviewed and approved by the Qazvin University of Medical Sciences (IR.QUMS.REC.1400.225).

## Data Availability

The data that support the findings of this study are openly available in the Harvard Dataverse at https://dataverse.harvard.edu/dataset.xhtml?persistentId=doi:10.7910/DVN/SQJFVA, reference number https://doi.org/10.7910/DVN/SQJFVA.

## APPENDIX A UNIDENTIFIED MODEL RESULTS

### Variable counts

**Table:**

Number of variables in your model	39
Number of observed variables	19
Number of unobserved variables	20
Number of exogenous variables	22
Number of endogenous variables	17

### Parameter summary

**Table:**

	Weights	Covariances	Variances	Means	Intercepts	Total
Fixed	20	0	0	18	2	40
Labelled	0	0	0	0	0	0
Unlabelled	22	10	22	4	15	73
Total	42	10	22	22	17	113

### Models

**Default model (default model)**

**Notes for model (default model)**

### Computation of degrees of freedom (default model)

**Table:**

Number of distinct sample moments	209
Number of distinct parameters to be estimated	73
Degrees of freedom (209–73)	136

### Result (default model)

The model is probably unidentified. To achieve identifiability, it will probably be necessary to impose 1 additional constraint.

### Default model

**Estimates (default model)**

**Scalar estimates (default model)**

The (probably) unidentified parameters are marked.

### Regression weights (university—default model)

**Table:**

mentall <‐‐‐ accommodation	unidentified
mentall <‐‐‐ gender	unidentified
SleepS <‐‐‐ accommodation	unidentified
SleepS <‐‐‐ gender	unidentified
SleepS <‐‐‐ age	unidentified
mentall <‐‐‐ age	unidentified
mentall <‐‐‐ HELIAA	unidentified
SleepS <‐‐‐ HELIAA	unidentified
SHI1 <‐‐‐ SleepS	
SHI2 <‐‐‐ SleepS	
SH3 <‐‐‐ SleepS	
D1 <‐‐‐ mentall	
D2 <‐‐‐ mentall	
D3 <‐‐‐ mentall	
D4 <‐‐‐ mentall	
D5 <‐‐‐ mentall	
D6 <‐‐‐ mentall	
D7 <‐‐‐ mentall	
DMM <‐‐‐ HELIAA	
APPP <‐‐‐ HELIAA	
ATL <‐‐‐ HELIAA	
UND <‐‐‐ HELIAA	
Reading <‐‐‐ HELIAA	
mentall <‐‐‐ SleepS	unidentified
SleepS <‐‐‐ mentall	unidentified

### Means (default model)

**Table:**

age
gender
accommodation
education

### Intercepts (default model)

**Table:**

SHI1
SHI2
SH3
D1
D2
D3
D4
D5
D6
D7
DMM
APPP
ATL
UND
Reading

### Covariances (default model)

**Table:**

gender <‐‐> age
accommodation <‐‐> age
accommodation <‐‐> gender
accommodation <‐‐> HELIAA
gender <‐‐> HELIAA
age <‐‐> HELIAA
education <‐‐> HELIAA
education <‐‐> accommodation
education <‐‐> gender
education <‐‐> age

### Variances (default model)

**Table:**

HELIAA	
accommodation	
gender	
age	
e45	unidentified
e46	unidentified
e34	
e35	
e36	
e37	
e38	
e39	
e40	
e41	
e42	
e43	
e47	
e48	
e49	
e50	
e51	
education	

### Minimization history (default model)

**Table:**

Iteration	Negative eigenvalues	Condition #	Smallest eigenvalue	Diameter	*F*	NTries	Ratio
0	e8		−4.258	9999.000	175,604.601	0	9999.000
1	e10		−0.399	1.619	94,770.919	17	0.419

### Model fit summary

#### CMIN

**Table:**

Model	NPAR	CMIN	DF	P	CMIN/DF
Saturated model	209	0.000	0		
Independence model	19	104,420.755	190	0.000	549.583

#### Baseline comparisons

**Table:**

Model	NFI Delta1	RFI rho1	IFI Delta2	TLI rho2	CFI
Saturated model	1.000		1.000		1.000
Independence model	0.000	0.000	0.000	0.000	0.000

#### Parsimony‐adjusted measures

**Table:**

Model	PRATIO	PNFI	PCFI
Saturated model	0.000	0.000	0.000
Independence model	1.000	0.000	0.000

#### NCP

**Table:**

Model	NCP	LO 90	HI 90
Saturated model	0.000	0.000	0.000
Independence model	104,230.755	103,171.338	105,296.450

#### FMIN

**Table:**

Model	FMIN	F0	LO 90	HI 90
Saturated model	0.000	0.000	0.000	0.000
Independence model	10.684	10.664	10.556	10.773

#### RMSEA

**Table:**

Model	RMSEA	LO 90	HI 90	PCLOSE
Independence model	0.237	0.236	0.238	0.000

#### AIC

**Table:**

Model	AIC	BCC	BIC	CAIC
Saturated model	418.000	418.857		
Independence model	104,458.755	104,458.833		

#### ECVI

**Table:**

Model	ECVI	LO 90	HI 90	MECVI
Saturated model	0.043	0.043	0.043	0.043
Independence model	10.687	10.579	10.796	10.687

#### HOELTER

**Table:**

Model	HOELTER .05	HOELTER .01
Independence model	21	23

### Execution time summary

**Table:**

Minimization	0.005
Miscellaneous	1.474
Bootstrap	0.000
Total	1.479
